# The Death of Dr. Virgil O. Hardon

**Published:** 1904-03

**Authors:** 


### EDITORIAL NOTES AND COMMENTS.

The Business office of THE JOURNAL-RECORD is No. 1007-1008 Century Building.

The Editorial office is 318-319-320 The Prudential.

Address all Business communications to Dr. M. B. Hutchins, Mgr.

Make remittances payable to THE ATLANTA JOURNAL-RECORD OF MEDICINE.

On matters pertaining to the Editorial and Original Communications address Dr. Bernard Wolff, Atlanta.

Reprints of original articles will be furnished at cost price. Requests for the same should always be made *on the manuscript.*

We will present, post-paid, on request, to each contributor of an original article, twenty (20) marked copies of THE JOURNAL-RECORD containing such article.

### THE DEATH OF DR. VIRGIL O. HARDON.

DR. VIRGIL ORVILLE HARDON died at his residence, 283 Peachtree street, Atlanta, on Sunday, February 7. The cause of his death was grip, with cardiac and pulmonary complications. Dr. Hardon was fifty-four years of age. He was born in Massachusetts and received his literary education at Brown University, Providence, R. I. He pursued his medical studies at Harvard University, and graduated from Bellevue in 1874. He came South twenty years ago, and for a time was engaged in business pursuits, but subsequently resumed the practice of medicine and confined his work to obstetrics and gynecology. He soon established a reputation and ultimately achieved a position in his specialty second to none in the State. For a number of years he had filled with honor the chair of obstetrics and gynecology in the Atlanta Medical College, and later in the conjoint institution known as the Atlanta College of Physicians and Surgeons. He possessed a mind of rare excellence, and his love for reading and enthusiasm for his profession led up to a degree of mental training and culture coupled with a skill and acumen in his chosen province of medicine which placed him among the first of his kind. As a writer and lecturer this mental training was especially noticeable. He had a singular facility for recognizing and seizing upon the strong points of a subject and presenting them in a way that was absolutely pellucid in expression and convincing in effect. As a teacher, he was pre-eminent, as is abundantly manifested by the lasting influence which he exerted upon the student body at the institution with which he was connected. No one was more frequently quoted by the students and no one was accounted more helpful to them than Dr. Hardon.

Socially, no man in the city enjoyed the respect and confidence of the public and the love and esteem of his fellow physicians more than did he. Blessed with a genial disposition, an inexhaustible wit and humor, and withal a cordiality and friendliness of manner, he won for himself true friends and retained them steadfast through all the sibilant whispers of scandal and the voice of calumny.

Among those thrown with him intimately as friend and companion he was the prince of good fellows. He was the life of any social gathering, his flow of spirits, his cheerfulness and geniality made the atmosphere surrounding him luminous and alluring. For such the realization will come slowly that the light in those mirthful eyes is forever dim, the merry, rallying voice forever silent, and the active, useful hands at rest.

"Here's rosemary, that's for remembrance, and here is pansies, that's for thoughts."

Dr. Hardon was several times married. He leaves a widow who was Miss Florence Talbot, of Elberton, whom he married in the spring of 1903, and who was soon to give to him what had long been his heart's dearest wish—an heir. But he died childless.

Enshrined forever in the hearts of his friends are the noble, manly and generous traits of their departed companion, while his victorious death seems to invite them to prepare to meet him in a region of eternal and undiminished happiness beyond the grave.

